# The effect of revascularization on recovery of mitochondrial respiration in peripheral artery disease: a case control study

**DOI:** 10.1186/s12967-021-02908-0

**Published:** 2021-06-04

**Authors:** Alexandra Gratl, Dominik Pesta, Leonhard Gruber, Fiona Speichinger, Ben Raude, Safwan Omran, Andreas Greiner, Jan Paul Frese

**Affiliations:** 1 Department of Vascular Surgery, Charité–Universitätsmedizin Berlin, Hindenburgdamm 30, 12200 Berlin, Germany; 2 Department of Vascular Surgery, Medical University of Innsbruck, Innsbruck, Austria; 3 Institute for Clinical Diabetology, German Diabetes Center, Leibniz Institute for Diabetes Research, Heinrich Heine University, Düsseldorf, Germany; 4 German Center for Diabetes Research (DZD), München-Neuherberg, Germany; 5 Department of Sports Science, Medical Section, Innsbruck, Austria; 6 German Aerospace Center, Institute of Aerospace Medicine, Cologne, Germany; 7 Department of Radiology, Medical University of Innsbruck, Innsbruck, Austria

**Keywords:** Mitochondrial function, Mitochondrial respiration, Peripheral arterial disease, PAD, Revascularization, Intermittent claudication

## Abstract

**Background:**

Peripheral arterial disease (PAD) is accompanied by myopathy characterized by mitochondrial dysfunction. The aim of this experimental study was to investigate the effect of revascularization procedures on mitochondrial function in ischemic and non-ischemic muscle.

**Methods:**

Muscle biopsies from patients with symptomatic stage IIB/III PAD caused by isolated pathologies of the superficial femoral artery were obtained from muscle regions within the chronic ischemic muscle (gastrocnemius) and from non-ischemic muscle (vastus lateralis) before and 6 weeks after invasive revascularization. High-resolution respirometry was used to investigate mitochondrial function and results were normalized to citrate synthase activity (CSA). Results are given in absolute values and fold over basal (FOB).

**Results:**

Respiratory states (OXPHOS (*P*) and electron transfer (*E*) capacity) normalized to CSA decreased while CSA was increased in chronic ischemic muscle after revascularization. There were no changes in in non-ischemic muscle. The FOB of chronic ischemic muscle was significantly higher for CSA (chronic ischemic 1.37 (IQR 1.10–1.64) vs. non-ischemic 0.93 (IQR 0.69–1.16) p = 0.020) and significantly lower for respiratory states normalized to CSA when compared to the non-ischemic muscle (*P* per CSA chronic ischemic 0.64 (IQR 0.46–0.82) vs non-ischemic 1.16 (IQR 0.77–1.54) p = 0.011; *E* per CSA chronic ischemic 0.61 (IQR 0.47–0.76) vs. non-ischemic 1.02 (IQR 0.64–1.40) p = 0.010).

**Conclusions:**

Regeneration of mitochondrial content and function following revascularization procedures only occur in muscle regions affected by malperfusion. This indicates that the restoration of blood and oxygen supply are important mediators aiding mitochondrial recovery.

**Supplementary Information:**

The online version contains supplementary material available at 10.1186/s12967-021-02908-0.

## Introduction

Followed by heart attack and stroke, peripheral arterial disease (PAD) is the third leading cause of arteriosclerotic morbidity [1]. Arteriosclerotic lesions lead to a chronic stenosis of arteries resulting in flow-limiting pathologies with a reduction of blood and oxygen supply in affected muscle regions. The leading clinical symptom in PAD, intermittent claudication, results in premature fatigue and reduced walking performance with a reduction of quality of life [2]. Depending on clinical manifestations and morphology of underlying arteriosclerotic lesions, conservative (medical therapy, life-style modification, exercise training) as well as interventional treatment (open surgical techniques, endovascular revascularization) is indicated [3]. As previously described, affected muscle regions of patients suffering from PAD are characterized by myopathy including histomorphological as well as metabolic alterations with mitochondria playing a crucial role in these pathophysiological mechanisms. Due to an insufficient blood supply, altered mitochondrial function and content may lead to additional oxidative stress resulting in subsequent myopathy and a vicious circle of ischemia–reperfusion injury [4–7]. High-resolution respirometry (HRR) is the gold standard method to evaluate mitochondrial function in vitro. The advantage of this method is that experiments are performed under biochemically controlled conditions with exclusion of any limitations caused by reduced oxygen supply during the assay due to flow-limiting arteriosclerotic lesions [4]. Results from HRR are commonly normalized to a mitochondrial marker in order to obtain information about changes of mitochondrial-specific respiration. Several markers are being used in the research setting, including individual mitochondrial proteins, mitochondrial DNA and specific phospholipids, such as cardiolipin [8]. Citrate synthase activity has been shown to be in good agreement with results obtained by the gold standard method of transmission electron microscopy [9]. In a previous study, we were able to demonstrate recovery of mitochondrial content and respiration six weeks after successful revascularization of underlying arteriosclerotic lesions. Gastrocnemius muscle biopsies from patients suffering from isolated flow-limiting pathologies of the superficial femoral artery (SFA) and a non-ischemic control group have been investigated by HRR [10]. Successful revascularization of underlying arteriosclerotic lesions resulted in restoration of blood flow and oxygen supply to the gastrocnemius muscle. As the leading symptom of PAD—intermittent claudication with pain-induced limitation of maximal walking distances—was successfully treated as well, also walking performance of patients improved. However, it remains unclear, whether the observed changes were caused solely by the restoration of blood flow or by an increased metabolic demand resulting from increased physical activity. The aim of this study was therefore to examine in isolation the impact of successful revascularization of underlying arteriosclerotic lesions on mitochondrial content and respiration. We hypothesized, that the restoration of blood flow is the major driver for mitochondrial recovery. To this end, mitochondrial content and function in chronic ischemic muscle regions (gastrocnemic muscle) have been compared to those from non-ischemic muscle regions (lateral vastus muscle) in patients with isolated lesions of the SFA. In the sense of translational medicine, our study integrates molecular findings to improve our understanding of normal vs. disease states in order to provide clinicians with optimized therapeutic approaches based on our laboratory data. Improved understanding of the pathophysiology of PAD will help to decide treatment options best suitable for the individual patient.

## Methods

### Ethics

All procedures were in accordance with the ethical standards of the institutional research committee (Charité`s Ethics Committee, Universitätsmedizin Berlin; Number of proposal EA4/021/16) and with the 1964 Helsinki declaration and its later amendments. Informed consent was obtained from all participants included in the study.

### Study participants

Ten patients with symptomatic PAD (Fontaine stage IIb/III) due to an isolated flow-limiting pathology of the superficial femoral artery (SFA) were consecutively recruited at the outpatient clinic of the Department of Vascular Surgery at the University Hospital Berlin, Charité. Patients with flow-limiting arteriosclerotic lesions of the infrarenal aorta, the iliac arteries as well as the deep femoral artery, with a stage IV PAD or with an allergy to local anaesthesia have been excluded from the study. In all patients, invasive revascularization of reasonable arteriosclerotic lesions for treatment of PAD was indicated and each case has been discussed individually within an interdisciplinary team to decide whether open or endovascular treatment should be performed.

Detailed medical history including demographic and anthropometric data (age, sex, body-mass index) and the presence of cardiovascular risk factors (arterial hypertension, diabetes mellitus, hyperlipidemia and smoking status) was obtained. Physical examination included pulse status, ankle brachial index (ABI) measurement and evaluation for trophic lesions of the lower extremities. Duplex ultrasound of the arterial tree of the affected leg was performed and for evaluation of maximal walking distances a treadmill test was performed at a continuous speed of 3 kms per hour and inclination of 0%. A successful completion of a walking distance of 500 m without the occurrence of intermittent claudication indicated that the patient did not suffer from any relevant walking induced pain causing a limitation of walking ability.

### Proof of technical success of performed revascularization procedure

Six weeks after vascular intervention, evaluation of hemodynamic parameters with measurement of ABI as well as treadmill tests were repeated. Ultrasound imaging of the treated lesion to confirm patency and to exclude relevant hemodynamic restenosis within the area of the revascularization procedure was performed.

### Muscle biopsy procedure

According to the inclusion criteria of isolated arteriosclerotic lesions of the SFA being reasonable for symptomatic PAD, the gastrocnemius muscle was considered as chronic ischemic muscle region and the lateral vastus muscle was considered as non-ischemic muscle region. Percutaneous muscle biopsies were taken using the modified Bergstrom percutaneous biopsy technique (Bergstrom Muscle Biopsy Needles, diameter 4.0 mm, Dixon Surgical Instruments, Essex, United Kingdom) [11, 12]. About 10–20 mg of muscle tissue was taken at each location. Muscle biopsies were obtained at the beginning of the vascular intervention (preoperative) and six weeks after successful revascularization (postoperative). Depending on the performed interventions, biopsies were taken under general or local anaesthesia using lidocaine. To prevent any possible effects of lidocaine on muscle mitochondrial function, infiltration of lidocaine into the muscle was avoided [13]. Muscle samples were transferred to ice-cold biopsy preservation solution (BIOPS) containing 10 mM Ca-EGTA buffer, 0.1 μM free calcium, 20 mM imidazole, 20 mM taurine, 50 mM 2-(N-morpholino)esthane-sulfonic acid hydrate, 0.5 mM dithiothreitol, 6.25 mM MgCl_2_, 5.77 mM ATP and 15 mM phosphocreatine (pH 7.1) and immediately transferred to our laboratory for further investigations.

### Permeabilization of muscle fibres

Details about the preparation of muscle fibres have been described elsewhere [14]. Permeabilization was completed chemically by incubation of fibres in 2 ml BIOPS containing 50 μg/ml saponin for 30 min. Followed by a 10 min period of washing in a mitochondrial respiration medium (MIR06) containing 110 mM D-sucrose, 60 mM K^+^-lactobionate, 0.5 mM EGTA, 3 mM MgCl_2_, 20 mM taurine, 10 mM KH_2_PO_4_, 20 mM HEPES, 1 g/l bovine serum albumin and 280 U/ml catalase, muscle samples with a wet weight of 1–3 mg were transferred to the respirometry chamber [14].

### High-resolution respirometry and measurement of mitochondrial respiration

HRR was performed using an Oxygraph-2 k (Orobros Instruments, Innsbruck, Austria) as described in detail elsewhere [14]. Briefly, the oxygraph consists of two closed chambers, both filled with 2 ml of MIR06. In the O2k, samples from the gastrocnemius muscle were directly compared to samples from the lateral vastus muscle in order to account for time-differences between the two respirometry assays. All measurements were performed in duplicates. A poloragraphic sensor within each chamber records the oxygen concentration and oxygen consumption continuously and mass-specific mitochondrial respiration is obtained as oxygen consumption per second, per milligram of wet weight of muscle tissue (pmol/(s*mg)). All measurements were performed at 37 °C within hyper-oxygenated chambers maintaining oxygen levels above > 250 mM to avoid any risk of potential oxygen limitations [14]. This was achieved by injecting gaseous oxygen into the open chamber prior to starting the measurements.

To investigate mitochondrial respiration, a substrate, uncoupler, inhibitor (SUIT) protocol was applied. LEAK state, mitochondrial respiration in the presence of substrates, but without ADP, was induced by the titration of 2 mM malate and 0.2 mM octanoylcarnitine (MOct_L_). After reaching steady state, 5 mM ADP was added to induce complex I-linked ADP-stimulated respiration (MOct_P_). With the titration of 5 mM pyruvate and 10 mM succinate, CI&II-linked respiration yields oxidative phosphorylation (OXPHOS) capacity (*P*). Stepwise addition (0.05 mM steps) of the protonophore carbonyl cyanide p-(triflouromethoxy) phenylhydrazone (FCCP) yields maximum mitochondrial respiration or electron transfer (*E*) capacity [10].

### Measurement of citrate synthase activity

Citrate synthase, an enzyme located in the mitochondrial matrix, is tightly associated with mitochondrial fractional area [9] and commonly serves as an established marker of mitochondrial content. About 10 mg of muscle tissue were initially stored at − 80 °C for further evaluation of CSA. After thawing, the samples were homogenized and protein concentrations of the lysates were determined using a QunatiPro™ BCA Assay Kit (Sigma-Aldrich, St. Louis, MO, United States of America). CSA was spectrophotometrically measured at 412 nm and 25 °C using a commercial Citrate Synthase Assay Kit (Sigma-Aldrich) according to the manufacturer’s instructions. Results from mass-specific mitochondrial respiration were normalized to CSA in order to obtain mitochondrial-specific respiration expressed as pmol/(s*CSA).

### Statistics

Statistical analysis was carried out in GraphPad Prism 8.1 (GraphPad Software Inc.; La Jolla, USA). Continuous data are given as mean ± standard deviation in case of Gaussian distribution or as mean with 95% confidence interval (in brackets) in case of a non-Gaussian distribution and are presented as box-plots including median and lower/upper quartiles; whiskers denote 5% and 95% percentiles. P-values < 0.05 were considered significant, p-values greater than < 0.10 were considered a trend.

Average age and sex frequency were calculated. To compare column means, a mixed-effects analysis in case of repeated measurements and a one-way ANOVA in case of non-repeated measurements was performed. A Holm-Sidak correction for multiple testing was performed in either case. The ratio of preoperative and postoperative values for CSA and all respiratory states normalized to CSA were calculated, results are given as absolute values and fold over basal (FOB). To achieve a normal distribution, data were transformed logarithmically (base 10) and column means were compared via a mixed-effect-analysis with a Geisser-Greenhouse correction with a Holm-Sidak correction for multiple testing was applied.

To exclude a bias of age and body-mass-index (BMI) on the assessed parameters, a linear regression analysis was carried out. Results are given as B-value ± standard error and p-value.

Based on improvements of mitochondrial function in PAD patients and age-matched controls [15, 16], a sample size calculation was performed under the assumption of an effect size (Cohen’s kappa) of 0.8, a type-1-error probability of 0.05 and power of 0.8. The resulting sample size was n = 10.

## Results

### Anthropometric and clinical data

Based on an a priori power calculation, ten patients were included into this study. Patients’ characteristics including presence of cardiovascular risk factors are given in Table 1. Detailed information about the clinical stage of PAD, morphology of SFA lesions and performed vascular interventions have been described earlier [10] and are shown in detail in Additional file 1: Table S1.

**Table 1:** Clinical data

	Preoperative	Postoperative	p-value
Age (years)	66.9 (IQR 61.5–73.4)	–	–
Sex (male/female)	–	–	–
Body mass index (kg/m^2^)	21.1 (IQR 18.3–24.1)	–	–
Cardiovascular risk factors (n)	2.5 (IQR 2.0–3.8)	–	–
Ankle brachial index (ABI)	0.65 (IQR 0.64–0.70)	0.92 (IQR 0.85–0.98)	< 0.001
Maximal walking distance (meters)	50 (IQR 32.5–50.0)	485 (IQR 207.5–500)	< 0.001

Overview of clinical data and comparison of pre- and postoperative ABI and maximal walking distances; Cardiovascular risk factors included arterial hypertension, diabetes mellitus, hyperlipidaemia and smoking status and are presented as number of risk factors present in patients; Data are expressed as median with IQR in brackets; A p-value of < 0.05 is considered to be significant

### Proof of technical success of vascular intervention

Vascular intervention lead to an increase of ABI in treated legs (p < 0.001) as well as an increase of maximal walking distances (p < 0.001) (Table 1). Clinical stage of PAD improved in all patients with stage IIB or III disease to a stage IIA disease [10]. All patients underwent ultrasound imaging and patency of arterial reconstruction was confirmed by exclusion of hemodynamic relevant restenosis in treated segments.

### Citrate synthase activity (CSA)

**Fig. 1:**
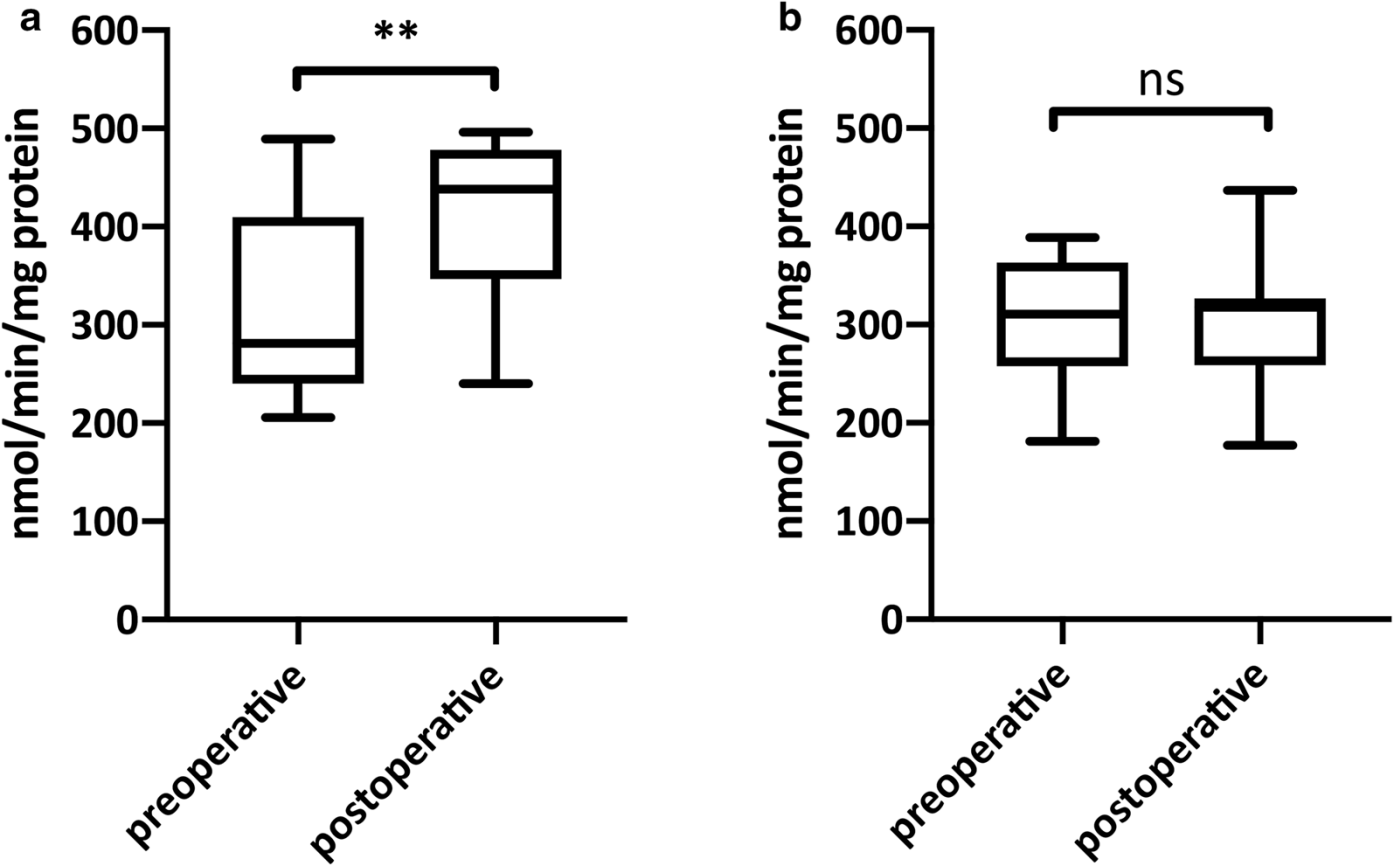
Citrate synthase activity in preoperative and postoperative samples; **a** chronic ischemic muscle; **b** non-ischemic muscle; a p-value < 0.05 was considered to be significant; **indicates statistical difference p < 0.01

Vascular intervention on reasonable SFA lesions lead to an increase of CSA as a proxy of mitochondrial content in chronic ischemic muscle regions (p = 0.010) (Table 2, Fig. 1). Non-ischemic muscle regions did not show any change of CSA between preoperative and postoperative state.

**Table 2:** Results from CSA, HRR and HRR normalized to CSA

	Chronic ischemic muscle (gastrocnemius muscle)			Non-ischemic muscle (lateral vastus muscle)		
	Preoperative	Postoperative	p-value	Preoperative	Postoperative	p-value
CSA (nmol/min/mg protein)						
	281.4 (252.4–391.8)	438.5 (361.4–471.3)	0.010	310.6 (266.8–357.4)	317.6 (278.3–324.1)	0.940
Mitochondrial respiration [pmol/(s*mg)]						
MOct_L_	16.1 (13.7–18.4)	13.7 (12.2–16.1)	0.575	16.5 (14.1–19.4)	18.3 (16.3–19.9)	0.169
MOct_P_	24.4 (20.9–29.7)	20.7 (17.5–22.5)	0.286	26.7 (23.4–29.4)	29.2 (23.3–33.7)	0.205
*P*	66.6 (57.8 –78.3)	53.2 (49.8–52.1)	0.006	64.2 (54.2–67.4)	60.2 (54.3–73.2)	0.341
* E*	75.9 (59.0–85.1)	59.1 (46.7–64.8)	0.002	69.5 (59.5–75.8)	65.7 (55.7–76.0)	0.617
Mitochondrial respiration [pmol/(s*mg)] per CSA						
MOct_L_	0.048 (0.040–0.074)	0.036 (0.031–0.040)	0.156	0.049 (0.048–0.053)	0.059 (0.049–0.078)	0.176
MOct_P_	0.072 (0.060–0.120)	0.051 (0.040–0.063	0.169	0.079 (0.078–0.083)	0.094 (0.079–0.110)	0.326
* P*	0.218 (0.196–0.266)	0.132 (0.166–0.150)	0.007	0.185 (0.173–0.230)	0.213 (0.171–0.266)	0.537
* E*	0.230 (0.195–0.279)	0.129 (0.120–0.154)	0.005	0.211 (0.180–0.252)	0.196 (0.176–0.243)	0.940

Results from CSA, HRR and HRR normalized to CSA; Data are expressed as median with IQR in brackets; A p-value < 0.05 is considered to be significant; *CSA* Citrate synthase activity, *MOct*_*L*_ maximal respiration after titration of malate and octanoyl, *MOct*_*P*_ maximal respiration after addition of ADP, *P* maximal respiration after addition of pyruvate and succinate, *E* maximal respiration after addition of FCCP

### Mitochondrial function

**Fig. 2:**
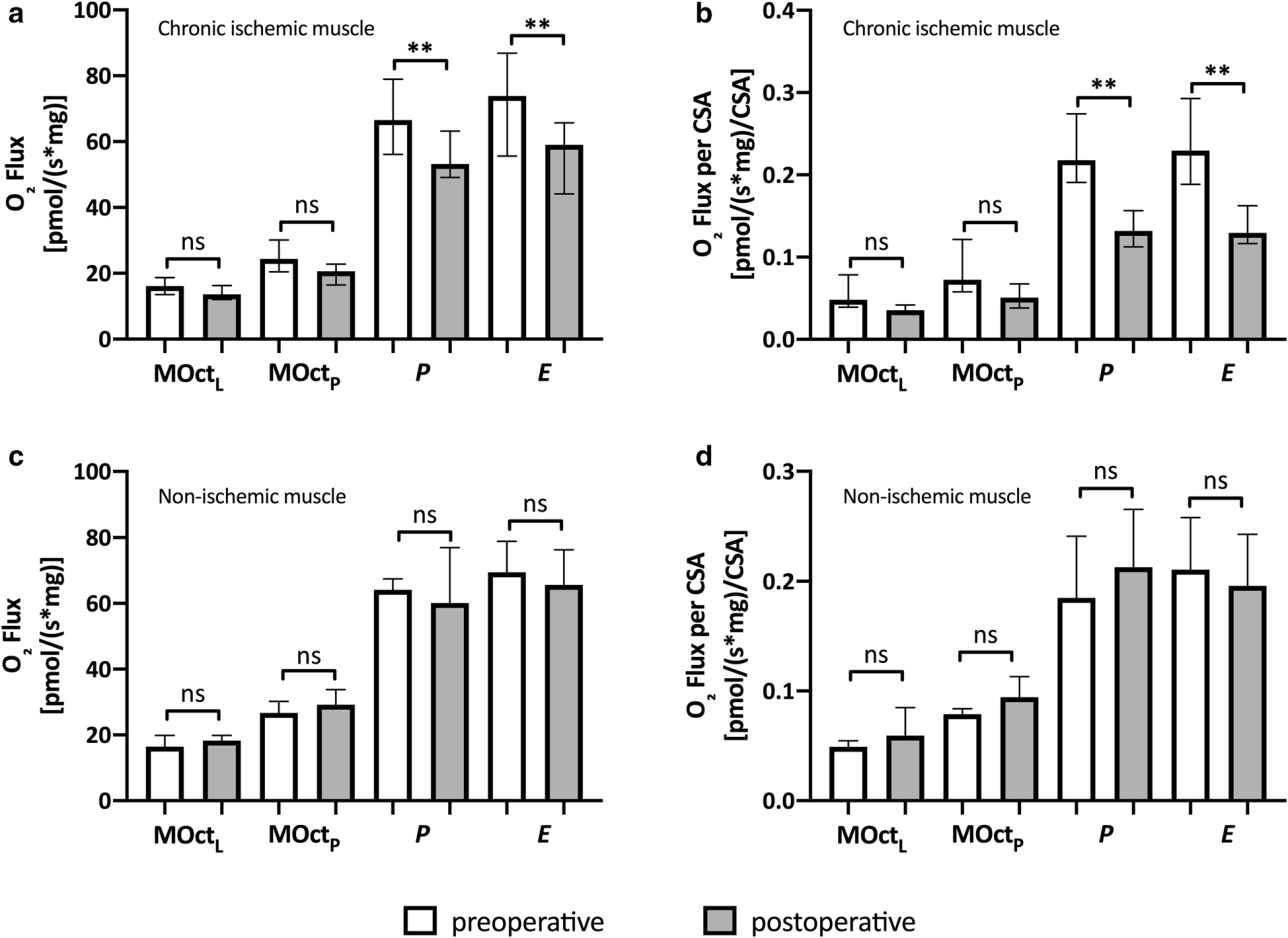
Results from respirometry in preoperative and postoperative samples in chronic ischemic and non-ischemic muscle; **a** results from high resolution respirometry expressed as oxygen (O_2_) flux per mg wet weight [pmol/(s*mg)] in chronic ischemic muscle; **b** results from high resolution respirometry normalized to results from citrate synthase activity (CSA) expressed as (O_2_) flux per CSA [pmol/(s*mg)/CSA] in chronic ischemic muscle; **c** results from high resolution respirometry expressed as oxygen (O_2_) flux per mg wet weight [pmol/(s*mg)] in non-ischemic muscle; **d** results from high resolution respirometry normalized to results from citrate synthase activity (CSA) expressed as (O_2_) flux per CSA [pmol/(s*mg)/CSA] in non-ischemic muscle; *ns* not significant; a p-value < 0.05 was considered to be significant; **indicates statistical difference p < 0.01; *CSA* Citrate synthase acitivity, *MOct*_*L*_ maximal respiration after titration of malate and octanoyl, *MOct*_*P*_ maximal respiration after addition of ADP, *P* maximal respiration after addition of pyruvate and succinate, *E * maximal respiration after addition of FCCP

HRR in chronic ischemic muscle regions showed a decrease of mitochondrial respiration in *P* (p = 0.006) and *E* (p = 0.002) after successful treatment of SFA lesions (Table 2, Fig. 2a and c). In non-ischemic muscle regions, all defined respiratory states remained unaltered in preoperative and postoperative muscle biopsies.

### Mitochondrial function normalized to CSA

Mitochondrial respiration normalized to CSA decreased for *P* (p = 0.007) and *E* (p = 0.005) after successful treatment of SFA lesions (Table 2, Fig. 2b and d). In non-ischemic muscle regions, there was no difference between preoperative and postoperative muscle samples regarding all defined respiratory states.

### Comparison of preoperative and postoperative values in ischemic and non-ischemic muscle regions (Fold over basal)

**Fig. 3:**
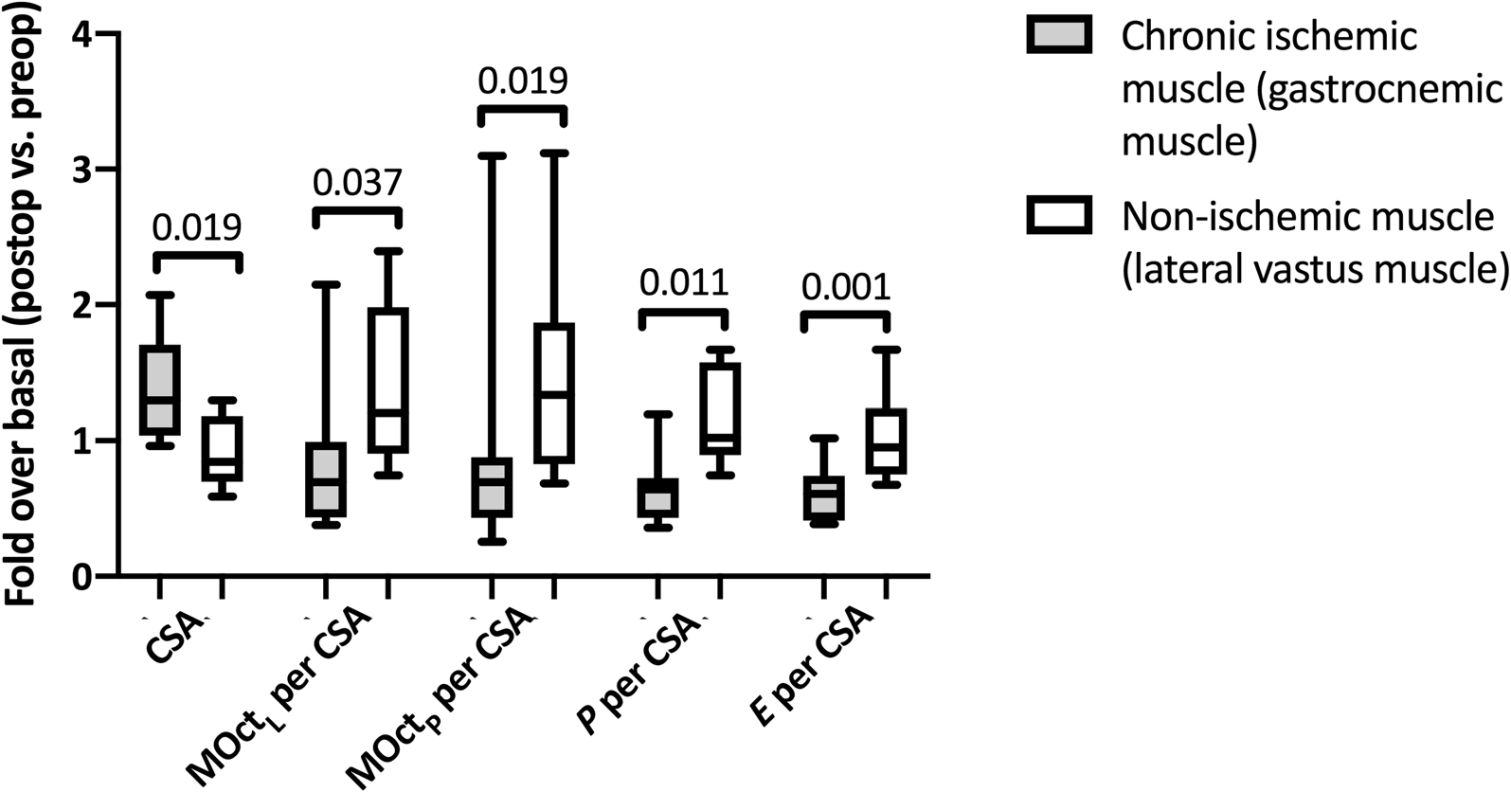
Fold over basal (FOB) in affected and unaffected muscle; a p-value < 0.05 was considered to be significant; *CSA* Citrate synthase acitivity, *MOct*_*L*_ maximal respiration after titration of malate and octanoyl, *MOct*_*P*_ maximal respiration after addition of ADP, *P* maximal respiration after addition of pyruvate and succinate, *E* maximal respiration after addition of FCCP

Preoperative results from CSA and respiratory states normalized to CSA where compared to postoperative measurements and FOB was calculated for chronic ischemic as well as non-ischemic muscle regions. The FOB of CSA in chronic ischemic muscle regions was higher when compared to the FOB in non-ischemic muscle regions (p = 0.019). FOBs of all respiratory states normalized to CSA in chronic ischemic muscle regions were lower when being compared to those from non-ischemic muscle regions (MOct_L_ p = 0.037; MOct_P_ p = 0.019; *P* p = 0.011; *E* p = 0.001) (Table 3 and Fig. 3).

**Table 3:** Fold over basal (FOB) in chronic ischemic and non-ischemic muscle

Parameter	FOB in chronic ischemic muscle (gastrocnemius muscle)	FOB in non-ischemic muscle (lateral vastus muscle)	p-value
CSA (nmol/min/mg protein)	1.37 (1.10–1.64)	0.93 (0.69–1.16)	0.020
MOct_L_ per CSA	0.83 (0.44–1.22)	1.39 (0.74–2.05)	0.037
MOct_P_ per CSA	0.87 (0.28–1.45)	1.47 (0.56–2.37)	0.020
*P* per CSA	0.64 (0.46–0.82)	1.16 (0.77–1.54)	0.011
*E* per CSA	0.61 (0.47–0.76)	1.02 (0.64–1.40)	0.010

Fold over basal (FOB) in chronic ischemic and non-ischemic muscle; Data are expressed as median with IQR in brackets; A p-value < 0.05 is considered to be significant; *CSA *Citrate synthase activity, *MOct*_*L*_ maximal respiration after titration of malate and octanoyl; *MOct*_*P*_ maximal respiration after addition of ADP, *P* maximal respiration after addition of pyruvate and succinate; *E* maximal respiration after addition of FCCP

## Discussion

With this study, we were able to confirm our hypothesis, that the restoration of blood flow and oxygen supply by successful revascularization of underlying arteriosclerotic lesions has a relevant impact on recovery of mitochondrial function by comparing results from chronic ischemic to non-ischemic muscle regions.

In patients with symptomatic PAD, flow-limiting pathologies of the arteries lead to a reduced blood flow and oxygen supply, resulting in unmet energy demands of the chronic ischemic muscle and concomitant cellular damage [17]. Repeating cycles of ischemia and reperfusion as a result of a limited blood flow to chronic ischemic muscle regions in PAD patients have been described to be associated with mitochondrial alterations [17, 18]. Recent results from studies inducing ischemia–reperfusion injury in animals specified the observed defects within the mitochondrial respiratory chain to complexes I, II and IV [19, 20]. In these studies, acute ischemia is induced using a tourniquet that is placed around the hind limbs and therefore the comparison to clinical manifestations of a chronic stage IIB or III PAD is difficult.

The relevance of mitochondriopathies in peripheral arterial disease has been tested in clinical studies [21] and our group was able to show in a previous study that mitochondria are able to recover six weeks after successful revascularization of reasonable arteriosclerotic lesions [10]. In this previous study, samples from the gastrocnemius muscle were investigated in patients with isolated arteriosclerotic lesions of the SFA compared to non-ischemic muscle of control persons. We demonstrated that i) CSA and therefore mitochondrial content is significantly increased after restoration of blood flow and ii) mitochondrial respiration decreased after the defined time period. Both these observations translated into a postoperative recovery of mitochondrial function when compared to non-ischemic healthy persons. This indicates an overall recovery of mitochondrial function after successful revascularization procedures [10]. But the underlying mechanism and contributing factors leading to mitochondrial recovery remains unclear. It has been shown that patients show an increased treadmill walking performance after revascularization procedures [22] and therefore an important factor that needs to be considered is habitual physical activity level of patients pre- and post-surgery. Mitochondria are able to adapt to increased metabolic demand such as exercise training by increasing mitochondrial function and enzyme activities [23, 24]. Increased metabolic demand results in fusion of a dynamic mitochondrial network, whereas a decrease in metabolic demand has been reported to result in fission and mitophagy [25–27]. One limitation of the current study is the use of only one marker to assess mitochondrial content. Although it is advantageous to assess a variety of markers, limitations in biological samples prevented inclusion of other mitochondrial markers in the current study.

Within the conception of the study, we took into account two main considerations. Firstly, the sole inclusion of patients with isolated pathologies of the SFA enable a differentiation of ischemic and non-ischemic muscle regions, allowing the identification of the underlying mechanism leading to mitochondrial recovery. Secondly, patients with relatively mild but symptomatic PAD have been included into this study. The inclusion of patients with stage IV PAD, who suffer from chronic pain related to ischemic wounds and delayed postoperative recovery, would have introduced a confounding factor. In our cohort, successful revascularization procedures lead to an increase of walking performance in all patients as intermittent claudication was dissolved or at least improved in all patients. As intermittent claudication likely excludes patients from physical activity participation due to walking-induced pain within chronic ischemic muscle regions, it has been hypothesized that patients suffering from symptomatic PAD are increasingly sedentary, resulting in impaired mitochondrial function of these patients [7]. After dissolving the intermittent claudication, patients will likely move more, which increases metabolic demand in chronic ischemic muscle regions after revascularization. It is therefore difficult to distinguish if the observed mitochondrial recovery was caused by the restoration of blood flow and oxygen supply or subsequent increased physical activity. By comparing results of chronic ischemic muscle regions to those of non-ischemic muscle regions, we were now able to demonstrate that the observed changes of mitochondrial function are likely mediated by the increased blood flow and oxygen delivery after successful revascularization of underlying arteriosclerotic lesions. Improved mitochondrial performance in ischemic gastrocnemius muscle after revascularization demonstrates that the restoration of blood and oxygen supply is likely a main driver. We reported previously that successful treatment of underlying arteriosclerotic lesions lead to an increase of mitochondrial content and a decrease of mitochondrial respiration. By comparing results to those from a heathy, non-ischemic control group we demonstrated that the postoperative decrease of mitochondrial respiration likely resembles a healthy physiological state. Our data suggest that mitochondrial flexibility allows for adjustment of mitochondrial function in the context of PAD with readjustment occurring after restoration of blood supply. Similar adaptation of hepatic mitochondrial metabolism have been observed in humans with non-alcoholic fatty liver disease [28].

Regarding the effect of increased physical activity on mitochondrial respiration, there are only a limited number of studies so far. Van Schaardenburgh et al. for example reported on PAD patients undergoing exercise training (calf raises and walking training). Patients were divided into a group of responders and non-responders in terms of walking performance after completing the exercise training for a predefined period of 8 weeks. Mitochondrial respiration was evaluated by the same method as in the present study and there were no changes in respiratory states within the group of positive responders [16]. These data are in line with our results as we demonstrate that–at least initially—an increased metabolic demand in terms of increased physical activity after dissolving intermittent claudication does not affect mitochondrial respiration in muscle regions with “normal” blood flow. Relevant arteriosclerotic lesions of arteries giving blood flow to the lateral vastus muscle have been excluded in our study population. We do not argue that physical activity and exercising is not able to influence mitochondrial function, but at this early stage, its significance is likely secondary. Walking training, as performed by our patients post-surgery, requires only about 3–4 metabolic equivalents (METs) and corresponds to about 50% of maximal oxygen uptake [29]. This intensity may simply be too low to impact mitochondrial function within this rather short time frame in our patients.

## Conclusion

Mitochondrial content and respiration are impaired in PAD patients. By interventional revascularization of underlying arteriosclerotic lesions and therefore restoration of blood flow and oxygen supply, mitochondrial function is able to recover. As pre- and postoperative mitochondrial function is unaltered in non-ischemic vastus lateralis muscle, we are able to ascribe the improved mitochondrial performance in ischemic gastrocnemius muscle to the successful revascularization rather than increased physical activity expressed by increased maximal walking distances due to the resolution of intermittent claudication.

## Supplementary Information

**Additional file 1: Table S1: **Clinical symptoms, morphology of SFA pathology and performed intervention; * SFA* superficial femoral artery; * TASC* Trans-Atlantic Inter-Society Consensus.

## Data Availability

The dataset generated and analysed during the current study is available from the corresponding author upon reasonable request.
