# Supplementary material for: The effect of revascularization on recovery of mitochondrial respiration in peripheral artery disease: a case control study
Source: J Transl Med. 2021 Jun 4;19:244. doi: 10.1186/s12967-021-02908-0 (PMC8178834; doi:10.1186/s12967-021-02908-0)
Supplement: Supplementary file 1 — Additional file 1: Table S1: Clinical symptoms, morphology of SFA pathology and performed intervention; SFA superficial femoral artery; TASC Trans-Atlantic Inter-Society Consensus. [file 12967_2021_2908_MOESM1_ESM.docx]

| **Nr** | **Age** | **Fontaine grade** | **TASC classification^37^** | **Type of vascular procedure** | **Details of vascular intervention** |
| --- | --- | --- | --- | --- | --- |
| **1** | 55.2 | IIB | A | endovascular | stent assisted angioplasty of the distal SFA |
| **2** | 73.8 | III | D | open surgery | above the knee femoropopliteal bypass |
| **3** | 61.3 | IIB | A | endovascular | stent assisted angioplasty of the distal SFA |
| **4** | 61.4 | IIB | B | hybrid procedure | endarterectomy of the origin of the SFA and stent assisted angioplasty of the distal SFA |
| **5** | 74.5 | IIB | D | open surgery | Endarterectomy of the origin of the SFA and embolectomy |
| **6** | 77.4 | IIB | C | open surgery | above the knee femoropopliteal bypass |
| **7** | 61.6 | IIB | B | hybrid procedure | endarterectomy of the origin of the SFA and stent assisted angioplasty of the distal SFA |
| **8** | 66.9 | IIB | A | endovascular | drug coated balloon angioplasty of the distal SFA |
| **9** | 66.9 | IIB | D | open surgery | above the knee femoropopliteal bypass |
| **10** | 72.2 | IIB | D | open surgery | above the knee femoropopliteal bypass |

Supplemental Table I - Clinical symptoms, morphology of SFA pathology and performed intervention; SFA – superficial femoral artery; TASC – Trans-Atlantic Inter-Society Consensus;
